# Advances in Titanium/Polymer Hybrid Joints by Carbon Fiber Plug Insert: Current Status and Review

**DOI:** 10.3390/ma15093220

**Published:** 2022-04-29

**Authors:** Michael C. Faudree, Helmut Takahiro Uchida, Hideki Kimura, Satoru Kaneko, Michelle Salvia, Yoshitake Nishi

**Affiliations:** 1 Faculty of Liberal Arts and Science, Tokyo City University, Yokohama-shi 224-8551, Japan; 2 Graduate School of Engineering, Tokai University, Hiratsuka-shi 259-1292, Japan; helmutuchida@tokai.ac.jp (H.T.U.); kimura@tokai-u.jp (H.K.); west@tsc.u-tokai.ac.jp (Y.N.); 3 Kanagawa Institute of Industrial Science and Technology (KISTEC), Ebina-shi 243-0435, Japan; satoru@kistec.jp; 4 Ecole Centrale de Lyon, CEDEX, 69134 Ecully, France; michelle.salvia@ec-lyon.fr

**Keywords:** hybrid joint, thermoplastic, thermoset, titanium, spot-welding, carbon fiber, electron beam

## Abstract

A literature review of up-to-date methods to strengthen Ti/carbon-fiber-reinforced polymer (CFRP) hybrid joints is given. However, there are little or no studies on Ti/CFRP joints by carbon fiber plug insert, which takes advantage of the extremely high surface adhesion area of ~6 μm CFs. Therefore, we cover the current status and review our previously published results developing hybrid joints by a CF plug insert with spot-welded Ti half-lengths to enhance the safety levels of aircraft fan blades. A thermoset Ti/CF/epoxy joint exhibited an ultimate tensile strength (UTS) of 283 MPa when calculated according to the rule of mixtures (RM) for the CF cross-section portion. With concern for the environment, thermoplastic polymers (TPs) allowed recyclability. However, a drawback is easy CF pull-out from difficult-to-adhere TPs due to insufficient contact sites. Therefore, research on a novel method of homogeneous low voltage electron beam irradiation (HLEBI) to activate a bare CF half-length prior to dipping in a TP resin was reviewed and showed that the UTS by the RM of Ti/**EB**CF/acrylonitrile butadiene styrene (ABS) and Ti/**EB**CF/polycarbonate (PC) joints increased 154% (from 55 to 140 MPa) and 829% (from 30 to 195 MPa), respectively, over the untreated sample. The optimum 0.30 MGy HLEBI prevented CF pull-out by apparently growing crystallites into the TP around the CF circumference, raising the UTS amount closer to that of epoxy.

## 1. Introduction and Background

Fiber-reinforced polymers (FRPs) have numerous applications, including airplanes, space vehicles, automobiles, sports equipment, ships, bridge cables, and wind turbines, to name a few [1,2,3,4,5], due to their formability, high strength, stiffness-to-weight ratios, and advanced fatigue and corrosion resistance [1,6]. Recently, for aircraft such as the Airbus A350 XWB and Boeing 787 Dreamliner, carbon-fiber-reinforced polymers (CFRPs) have reached about 50 wt.% and have been applied to the wings, as well as the fan blades, of turbo fan engines.

Titanium alloys, on the other hand, were first developed for the aerospace industry [7] and have since been utilized for jet engine components and airframe structure due to their high strength, stiffness, toughness, fatigue, and excellent resistance to corrosion [8] when coupled with CFRPs [9]. In addition, Ti alloys have the advantage of maintaining mechanical properties at high operating temperatures and can be used for structures requiring heavy loads such as wing–fuselage connections and landing gear [8]. Most commercial airlines have about 10 wt.% Ti; however, present modern aircraft such as the Airbus A350 and Boeing 787 are built with slightly greater than 10 wt.% [8]. Ti alloys are used for jet engine components with operation requirements of 673 to 773 K (400 to 500 °C) and can include low-pressure compressor parts, plug and nozzle assemblies in the exhaust section, and fan blades [8,10,11].

To prevent impact damage and fracture, a Ti sheath is often mechanically fitted, assembled, and mounted to the leading edge of CFRP fan blades, having the advantage of spontaneous adhesion with full contact at the Ti–epoxy interface.

Traditionally, fasteners have been used to join materials in aircraft. Although fasteners have the advantages of simple processing, high joining strength, and small scatter of data, disadvantages are increased in weight due to the fasteners themselves, and their sealing performance is low. Also, bolt holes reduce the cross-sectional area of the part and, along with threads, can act as stress concentrators [12]. Problems with drilling holes in FRP laminate composites include fiber breakage, peeling of top plies at the hole entry, resin degradation at the hole wall, and delamination of the laminate bottom plies [13,14], all of which can result in crack generation or propagation during fatigue [15].

However, the benefits of using adhesive joints are: (1) the sealing is complete; (2) a weight reduction from the absence of fasteners; (3) the avoidance of stress concentration from the fastener holes; (4) and an absence of drilling so that possible damage to the outer plies is avoided. Hence, adhesive joints typically have higher fatigue strength than bolted joints [15]. However, adhesive bonding has its disadvantages: selection is difficult when joining two different materials; additional steps are needed for degreasing and etching the joining surfaces to attain high adhesion strength; and chemically treated adhesive joints can degrade by oxidation in a few hours, decreasing the bonding strength [16]. Thus, it can be challenging to achieve strong adhesive joints. Adhesion alone can make aircraft fan blades more vulnerable to fracture at the Ti–CFRP interface by possible impact from bird strike or other airborne debris during flight, along with heterogeneous stresses generated by the strong airflow of jet engine compressors.

There are several studies found in the literature to create stronger Ti-CFRP joints; the most up-to-date are reviewed here to be useful to the reader. The methods include laser treatment, improving bolted joints, carbon fiber nanotubes (CNTs), anodizing, brazing, friction riveting, inductive heating, ultrasonic additive manufacturing, and novel bio-inspired adhesive.

Lately, the use of lasers has been a widely researched method finding success [17,18,19,20,21,22,23,24]. For Ti/short carbon fiber polyphenylene sulfide CFRP joints, laser welding was adopted, achieving a maximum tensile shear load of 2052 N at an optimum 700 W laser power [17]. For Ti/thermoplastic PEEK CFRP joints, a laser to the Ti made successful joining possible by creating a rough Ti surface for the molten PEEK resin to intricately flow into, along with the formation of a new phase of CTi_0.42_V_1.58_ at the Ti–PEEK interface [18]. Laser texturing found success: the texturing of a 0.2 mm wide grid on a Ti surface prior to hot pressing was reported to raise the maximum shear force of Ti/CFRP joints three times to 5286 N [19], while for a Ti/thermoplastic CFRP joint, laser texturing the Ti surface significantly increased wettability at the interface, raising the shear force 156% over the untreated sample [20]. The effect of the scanning speed of laser joining was investigated for a Ti/PEEK CFRP joint, revealing that higher scanning speeds created fewer defects on the Ti surface and less bubbles in the CFRP, along with mechanical interlocking and chemical bonding. For the Ti/PEEK CFRP joint, a scanning speed of 0.8 mm/min resulted in a maximum shear force of 1024 N [21]. The method of laser-riveting Ti pins to Ti parts followed by adhesive bonding and surface structuring of the Ti parts was demonstrated to improve mechanical fatigue life over that of conventional Ti/CFRP joints, along with higher stiffness with equal strength [22]. A metal surface laser plastic-covered technique with high-speed laser rotational welding technology was reported to substantially improve the shear strength and fatigue resistance of Ti/CFRTP joints by producing a hardened layer on the Ti surface. A fracture was reported to occur within the CFRTP but not the interface, demonstrating the strength of adhesion [23]. Another study reported that the pretreatment method of laser cleaning thermoset CFRP with laser plastic-covered processing of the Ti surface to create a thermoplastic coating generated mechanical interlocking coupled with chemical bonding for Ti/thermoset CFRP joints, enhancing strength [24].

There has been ample research to improve strength of bolted Ti/CFRP joints, and the most current are covered here [22,25,26,27,28,29,30]. Notable is a recent study of an innovative PEEK CFRP rivet cut from pultruded continuous fiber rods and heated directly into epoxy CFRP plates. The resulting joint had nearly twice the shear and tensile strengths than that of Ti bolts [25]. Also, hybrid bonded/bolted (HBB) joints have been increasingly utilized in the aerospace field due to their higher tensile properties [26]. For example, the HBB joints of three bolts aligned in the tensile direction with adhesive had higher tensile strength than those with one or two bolts and were higher than pure bolted or pure bonded joints [26]. A study of bolted Ti/CFRP joints reported a “dynamic installation” method that reduced typically undesirable damage to the top and bottom plies [27]. For single-lap Ti/polyimide (PI) lap joints, two types of Ti alloy inserts, bushing and embedded conical nut, were fabricated to repair the bearing damage zone [28]. The use of Ti rivets was coupled with a laser riveting process, as mentioned above, to strengthen Ti/CFRP joints [22].

There have been recent studies on the testing and analysis of deformation and fracture mechanisms of Ti/CFRP bolted joints [29,30]. For double-lap single-bolt Ti/CFRP joints, a damage model was constructed to characterize fitting tolerance effects on damage and failure during quasi-static loads [29]. For single-lap pinned Ti/CFRP joints, a dynamic test platform based on an electromagnetic loading technique was developed to analyze fracture mechanisms, demonstrating, as expected, the most damage in the CFRP [30].

The use of carbon fiber nanotubes (CNTs) has been gaining significant attention in strengthening Ti/CFRP joints. The flame method was utilized to deposit CNTs on Ti to enhance the resistance-welding of hybrid Ti/TP composite joints. The CNTs acted as “connectors”, increasing joint adhesion [31]. Reinforcing a PI matrix with multi-walled CNTs (MWCNTs) was reported to strengthen the Ti–PI interface of a Ti/PI multilayered alternating laminate joint [32], with MWCNT diameters from 2 to 20 nm, enhancing the interface mechanical properties [33]: the average diameter of 8 nm increased the interface mechanical performance almost 180% over that without MWCNTs [33]. A novel co-bonding process was reported joining epoxy MWCNT-reinforced CFRP with Ti to make a fiber metal laminate (FML) in which the joining of Ti to CFRP was performed simultaneously with the CFRP manufacturing. Adding MWCNTs to epoxy resin increased fracture resistance over 140%, while, for safety, making structural health monitoring (SHM) possible to locate crack propagation and stresses before joint fracture [34].

Research on anodizing the Ti surface prior to adhering to the CFRP includes the application to ultrasonic welding for Ti/Nylon-6 CFRTP lap joints [35]. Other studies include chromic acid anodization to adhere Ti to TP [36], as well as to the amorphous TPs of polyphenylquinoxaline, glass-filled Ultem polyetherimide, unfilled Ultem polyetherimide, and Victerex polyethersulfone of single lap joints [37]. The method of resin precoating (RPC) after anodizing, grinding, or acid-pickling of the Ti surface was investigated [38]. The study reported NaOH anodizing with an RPC treatment resulted in 22.0 MPa bond strength in single-lap shear tests, 105.3 and 70.1% higher than acid-pickled and ground, respectively [38].

In addition, a brazing method was utilized to fabricate Ti/short fiber PEEK C/C joints [39]. A metallic foam interlayer was introduced, producing a homogeneous microstructure, changed stress distribution, and enhanced mechanical properties of the joint [39].

In addition, for Ti/short fiber PEEK CFRP joints, employing a friction riveting process with fast rotation speed, friction time, and forging pressure had success, with high pull-out tensile strength ranging from 6.3 to 10.7 kN [40].

Another method, induction heating, was utilized for Ti/PPS (thermoplastic polyphenelene sulfide) tensile lap joints [41].

Ultrasonic additive manufacturing (UAM) was utilized to fabricate Ti/3-D CFRP structures, demonstrating that ultrasonic energy and surface roughness could yield increased shear strengths [42].

Finally, a mechanically novel technique of fabricating bio-inspired adhesive single-lap joints with a microstructural surface pattern resembling a gecko allowed fracture path controllability for lap joints [43].

However, none of these technologies applied an insert intricately embedded into both joint half-lengths for strong connection. The CF plug junction is employed, which is a cross-weave that can be simply set into a Ti half-length slit prior to spot welding. Despite the stress concentrators that can be generated at spaces within the weave, the weave pattern itself is advantageous, reducing flaw sensitivity and providing substantial mechanical property improvement of the composites [44]. Wavelength dispersive X-ray spectroscopy (WDS) analysis has shown that rapid spot-welding prior to rapid cooling solidifies Ti metal intricately between individual CFs in cross-weave CF plugs [10,11,45]. The advantages of spot beams are that the beam is highly focused, the energy is controlled precisely to allow rapid melting prior to rapid solidification, and the vacuum atmosphere protects molten metals from trace oxides and nitrides [10,11,46].

Therefore, to improve the adhesive force of the Ti–CFRP interface, we reviewed the literature background [10,11,45,46,47] of joining Ti with CF cross-weave plug inserts to take advantage of the extremely high surface areas of ~6 μm CFs for high adhesion.

It follows that the critical interface area (*S*_c_) to achieve the maximum tensile stress of a single CF implanted in polymer can be calculated by the following equation [47]:

*S*_c_ = π*rL* (1)

For CF in epoxy, *S*_c_ was experimentally determined to be 4.71 × 10^−8^ m^2^ when *r* and *L* were the CF radius (3 × 10^−6^ m) and length (5 × 10^−3^ m), respectively, for CF with an extremely high tensile strength (*σ*_b_) of 6 GPa (6 GN/m^2^) [47]. The reported results showed in single-fiber tests implanted into epoxy glue that a 5.0 mm implant length *L* apparently gave the highest CF tensile strengths in the range of 2.7 to 4.8 GPa, as opposed to an *L* below 2.5 mm giving 0.8 to 4.2 GPa. It was also reported in a single-fiber tensile test that, when the CF implant depth in epoxy glue was more than 5 mm, tensile strength, i.e., adhesion force at the median fracture probability *P*_f_ = 0.50, was ~4.0 GPa, which was more than that at a 1.5 mm depth (3.3 GPa) [47].

Thus, the critical resistant (shear) stress *σ*_c_ (MN/m^2^) to pull out a single fiber of CF from epoxy glue is a small friction resistance force of approximately 3.6 MPa calculated by [47]:

*σ*_c_ = [πr^2^/*S*_c_]*σ*_b_ = [d/2L]*σ*_b_ = 3.6 MPa (2)

Collectively, a high force of friction is produced between the high surface area of the 6 μm diameter CFs and epoxy resin, creating strong adhesion with full contact [46]. Thus, based on this concept, new Ti/CF/epoxy joint to improve the strength of Ti/epoxy by multiplying the contact area 450 times with a CF plug was innovated [46].

However, due to their crosslinked macromolecular structure, thermosets (TSs) are not easily recyclable; degradation and disposal pose significant problems for our environment. On the other hand, thermoplastic polymers (TPs) are highly desired over TSs since they can be melted and reformed for recyclability and sustainability, have shorter production times, lower moisture absorption, increase crack resistance, and lower material costs [10,11].

An example of a TP is ABS, constructed with rubber structure phase fine particles of the elastomer polybutadiene (PB: –(CH_2_–CH=CH–CH_2_)–) distributed in an amorphous phase matrix of acrylonitrile styrene (AS: –[(CH_2_–CH < –CN>)_n_ –(CH_2_–CH < –C_6_H_5_>)_m_]–). ABS has high crack resistance and recyclability, and it is only ~20% of the cost with ~10% of the solidification period of epoxies [10]. PC TP polymer is constructed of aromatic hard segments and carbonate groups (PC: –(O– < –C_6_H_4_ > –C(CH_3_)_2_– < –C_6_H_4_ > –OC=O–)_n_), is recyclable, and has strong resistance to impact and high temperature [11].

However, a disadvantage of TPs is low adhesive strength to CF due to easy fiber pull-out from the typically difficult-to-join TPs. While TS epoxy has strong interfacial adhesion with CF around the entire fiber circumference, in the TP nylon-6 CFRTP, for example, dendritic crystalline (hard segments) was found to grow incompletely and heterogeneously around the CF circumference [48]. The initial crystallites heterogeneously nucleated at sparse point contacts on the CF surface due to low wettability between the TP and CF. Hence, the ultimate tensile strength (UTS) has been found to be inadequate in fabricated Ti/CF/TP joints [10,11] in comparison to the full contact of TS epoxy [46].

The weak bonding between TPs and CF has also been attributed to CF lattice structure having graphitic basal planes with nonpolar surfaces and chemical inertness due to the manufacturing steps of high-temperature carbonization and graphitization [49,50,51]. Also, the surface smoothness, negligible adsorption characteristics, and lipophobicity of CF lead to insufficient bonding with matrix materials [52,53].

However, activation by applying a light electron (e–) charge with homogeneous low voltage electron beam irradiation (HLEBI) has been gaining attention, since it has been found to improve many materials [49,54,55,56,57,58,59,60,61,62,63,64], including: enhancing wetting and mist resistance [55,56,57,58]; increasing the impact value of polycarbonate polymer [63]; increasing the adhesion of glass fibers to polymers [59,60]; adhering the flat surfaces of metal/polymer joints such as Al/PU [61] and Cu/PU [62]; and the joining of 18-8 stainless steel and CFRP (18-8/CFRP) [54]. For an interlayered composite of three plies of carbon fibers between four layers of polypropylene sheets ([PP]_4_[CF]_3_), a 0.22 MGy HLEBI dose directly to carbon fibers in a N_2_ atmosphere to sized and 0.30 MGy to unsized carbon fibers prior to lamination assembly and hot pressing improved adhesion, raising the bending strength of the composites [PP]_4_[CF]_3_ and [PP]_4_[unsized CF]_3_ [48]. For [PP]_4_[unsized CF]_3_, when an HLEBI dose of 0.22 MGy was applied in an optimal 2000 ppm O_2_-rich atmosphere, the bending strength was raised for all the fracture probabilities over the untreated sample [65]. However, it is not recommended to use unsized carbon fibers in practical situations due to an inferiority in processing and strength compared to sized CFs.

HLEBI is a comparatively simple technique that does not use atoms, catalysts, or chemical treatments. Large platens can be treated.

HLEBI activates the CF surface by decreasing the density of naturally occurring dangling bonds in the hexagonal graphite structure detected by a reduction in electron spin resonance (ESR) peak height [66]. Applying HLEBI to CF has been reported to strengthen the CF itself, increasing fracture stress and elasticity, along with ductility [67,68].

Therefore, to increase the UTS of Ti/polymer joints, we present a research review of introducing a CF plug with a high connecting surface area of fine carbon fibers [10,11,46] and, for environmentally friendly Ti/TP joints, the activating of bare CF half-lengths by homogeneous electron beam low voltage irradiation (HLEBI) prior to dipping in a TP resin [10,11].

For simplicity, the joint designations will be (A) “Ti/polymer” for titanium/polymer joint spontaneous adhesion with no CF or glue; (B) “Ti/CF/polymer” for those with CF plug inserts; (C) “Ti/**EB**CF/polymer” or “Ti/**Ni**CF/epoxy” for those with a CF plug that is HLEBI (EB)-treated or a CF plug that is Ni-plated, respectively; and (D) “^c^Ti/CF/polymer”, “^c^Ti/**EB**CF/polymer”, or “^c^Ti/**Ni**CF/epoxy” with a superscript ‘c’ for those whose UTS was calculated for CF cross-section portions by the rule of mixtures.

**Figure 1:**
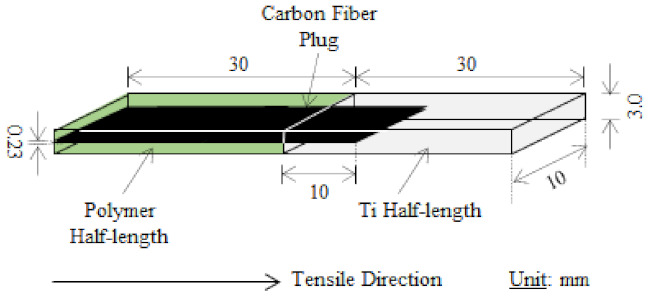
Schematic illustration of CF plug joint specimen [10,11,46].

A background of thermoset Ti/CF/epoxy joints with the same geometry as the TP joints illustrated in Figure 1 is included here to serve as a measure for more environmentally friendly TP joints to attain [46]. The ultimate aim is the developing and strengthening of Ti/CF/TP joints closer to or, if possible, beyond that of Ti/CF/TS epoxy joints with high concern for the environment and safety.

## 2. Introducing CF Plug for Increased UTS of Titanium/Polymer Joints

Figure 1 shows the schematic of a CF plug specimen with the following dimensions: total length, width, thickness, CF plug length, and CF plug thickness of 60, 10, 3.0, 40, and 0.23 mm, respectively. The length of the CF plug into the Ti and polymer is 30 and 10 mm, respectively. The Ti and polymer half-lengths are equal lengths at 30 mm each [10,11,46].

**Figure 2:**
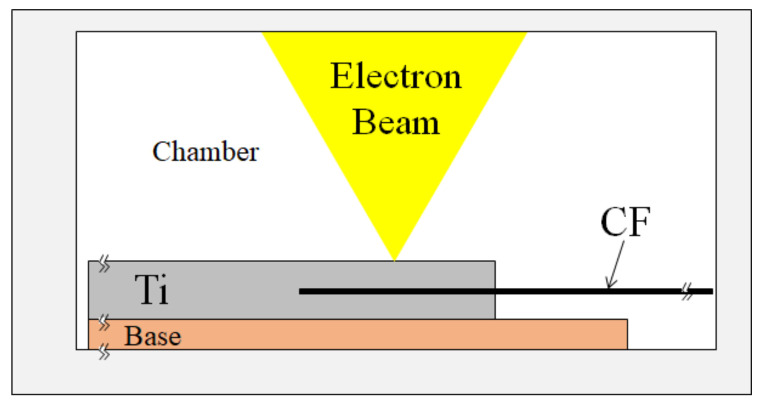
Schematic diagram of focused EB spot welding of CF with molten Ti under vacuum atmosphere.

The Ti/CF/polymer joints were constructed by taking advantage of the extremely high surface area of 6 μm CF cloth. To achieve this, a two-step process was employed:

**Step 1:** The Ti/CF half-length was assembled by spot welding, as illustrated in Figure 2 [10,11,46].

**Step 2:** The polymer/CF half-length was assembled by dipping the exposed CFs in molten thermoplastics such as ABS [10], PC [11], or thermoset epoxy [46].

### 2.1. Ti/CF Half-Length: Assembly by Spot Welding and Examination

The first step of rapid spot welding (Figure 2) used to contact and wrap the CF with molten Ti by capillary phenomenon is described here. The melting was performed by electron beam (EB) at a 10 kV potential and a 25 ± 5 mA current under a vacuum with 9.3 × 10^−4^ Pa residual gas pressure [10,11]. The EB welding process involved rapid heating above the Ti melting point (M.P.) of 1943 K (1670 °C) with a heating power term of 10 s followed by rapid solidification by heat sink, where the solidification point of saturated C- and O-rich Ti occurred below the Ti M.P.

### 2.2. Polymer/CF Half-Length: Assembly

The remaining half-length of exposed CF cloth was dipped in the polymer at a temperature above the melting point, dried, and solidified, resulting in the finished samples [10,11,46].

### 2.3. Tensile Testing and Analysis

The tensile tests were conducted with an Autograph tensile tester (Shimadzu Model AG-10TE: Shimadzu Corporation, Tokyo, Japan) at 1.0 mm/min at room temperature [10,11,46]. The stress–strain curves were recorded according to crosshead displacement and confirmed via recording by video. Because the polymer half-length deformed more than the Ti/CF during the tensile tests, true stress–strain curves could not be adaptable due to heterogeneous deformation. Therefore, the UTS σ_b_ (MPa) was obtained from the nominal stress–strain curves. Since the UTS of the joints was smaller than that of Ti or CFRP, slip could not be observed.

After the tensile tests, sample cross-sections perpendicular to the tensile testing direction were cut 5 mm deep into the Ti/CF half-length for analysis. Element mapping of C, Ti, titanium carbide (TiC), and titanium dioxide (TiO_2_) was carried out with an electron probe micro-analyzer (EPMA-1610, 15 kV, 10 nA/Shimazu, Kyoto, Japan). X-ray diffraction (XRD) (Cu-Kα, MiniflexII, Rigaku, Tokyo) was performed using a 10^−3^ deg/s scanning rate. Lattice structures of the compounds were determined by standard diffraction peaks evaluated by the ICDD (International Centre for Diffraction Data). For more detail, please refer to [10,11,46].

### 2.4. Results of Addition of CF Plug to Increase Tensile Stress of Ti/Polymer Joints

**Figure 3:**
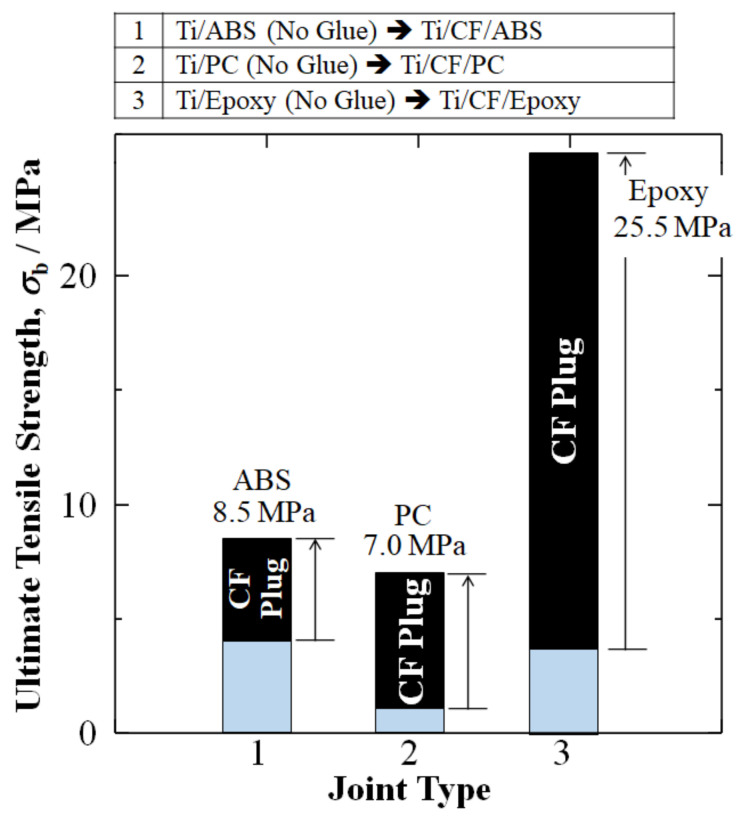
Improvements in ultimate tensile strength (UTS) *σ*_b_ (MPa) of Ti/Polymer joints by addition of CF plug. Data is from: Hasegawa, Faudree, Matsumuara, Jimbo, and Nishi (2016) [10]; Hasegawa, Faudree, Enomoto, Takase, Kimura, Tonegawa, Jimbo, Salvia, and Nishi (2017) [11]; and Nishi, Uchida, Faudree, Kaneko, and Kimura (2019) [46], for titanium joints with ABS, PC, and epoxy, respectively.

The initial attempts to employ CF plugs for Ti/polymer joints include Hasegawa et al. (2016), who fabricated Ti/CF/ABS [45], which paved the way for further developments [10,11,46]. Figure 3 summarizes our research results of CF plug addition to Ti/TPs (ABS and PC), along with a Ti/TS (epoxy). Without a CF plug, Ti/ABS, Ti/PC, and Ti/epoxy joints adhere spontaneously and have been experimentally found to have relatively low UTS amounts of 4.0, 1.0, and 3.5 MPa, respectively [10,11,46].

However, Figure 3 shows that, by the addition of a CF plug taking advantage of the broad interfacial surface area of *d* = 6 μm CF cross-weave cloth, UTS could be raised 2.1 times (+113%), 7 times (+600%), and 7 times (+630%) for the Ti/CF/ABS, Ti/CF/PC, and Ti/CF/epoxy joints to 8.5, 7.0, and 25.5 MPa, respectively [10,11,46].

## 3. Activating CF Plug with HLEBI to Increase UTS for Ti/CF/Thermoplastic Joints

Since UTS amounts of the TP joints Ti/CF/ABS and Ti/CF/PC have been reported to still be significantly lower than that of TS Ti/CF/epoxy, HLEBI was used to activate the bare carbon fiber surfaces of the TP joints prior to dipping in a TP to make Ti/**EB**CF/ABS and Ti/**EB**CF/PC joints.

The steps including HLEBI are as follows:

**Step 1:** Ti/CF half-length assembly by spot welding.

**Step 2:** The new part of the process is, after Ti solidification, the half-length of exposed CF of the Ti/CF joint sample is surface-activated on both sides by HLEBI.

**Step 3:** Polymer/CF half-length assembly.

### 3.1. HLEBI Method

Prior to dipping in the polymer, the remaining CF half-length was treated on both sides by an optimal 0.30 MGy homogeneous low voltage electron beam irradiation (HLEBI) or (EB) to fabricate Ti/**EB**CF/ABS and Ti/**EB**CF/PC joints. Repeated irradiations to both side surfaces of the samples were used to increase the total irradiation dose. The interval between the end of one irradiation period and the start of the next operation was 30 s. Details and parameters are given elsewhere in Hasegawa et al., (2016, 2017) [10,11].

### 3.2. Increase in UTS by HLEBI Activation

**Figure 4:**
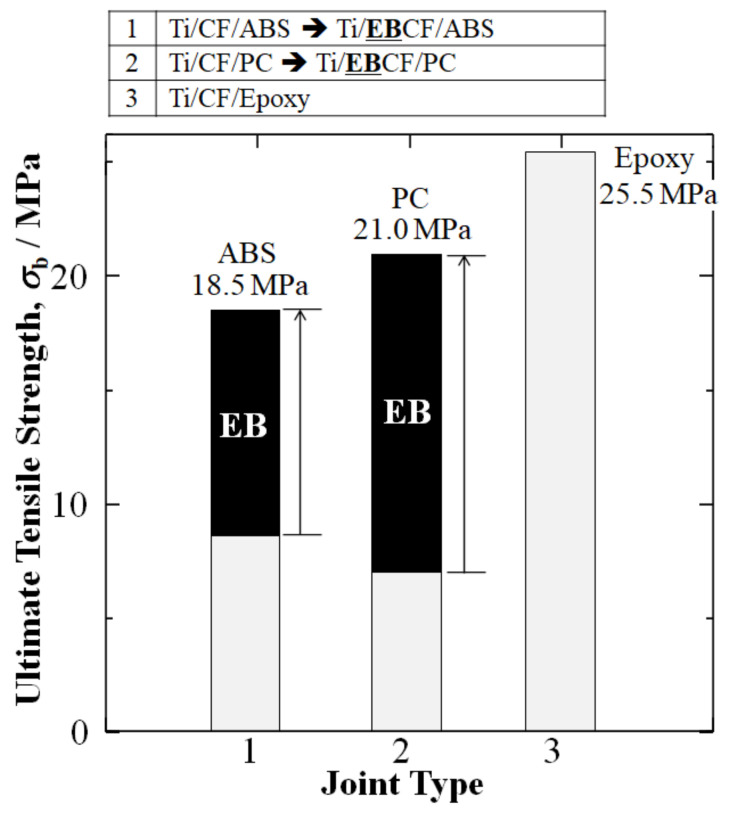
Improvements in ultimate tensile strength (UTS) *σ*_b_ (MPa) of Ti/TP joints by HLEBI activation of CF plug closer to that of TS epoxy. Data is from: Hasegawa, Faudree, Matsumuara, Jimbo, and Nishi (2016) [10]; Hasegawa, Faudree, Enomoto, Takase, Kimura, Tonegawa, Jimbo, Salvia, and Nishi (2017) [11]; and Nishi, Uchida, Faudree, Kaneko, and Kimura (2019) [46], for titanium joints with ABS, PC, and epoxy, respectively.

Figure 4 shows that, by applying HLEBI activation at 0.30 MGy to the exposed carbon fiber half-length prior to dipping in a molten thermoplastic resin, the UTS amounts of Ti/**EB**CF/ABS and Ti/**EB**CF/PC were increased 114% and 200% to 18.2 and 21.0 MPa, respectively, over those without HLEBI: Ti/CF/ABS and Ti/CF/PC of 8.5 and 7.0 MPa, respectively (Figure 4) [10,11]. The increase was closer to the goal of Ti/CF/epoxy (25.5 MPa) [46]. In addition, this was 4.6 times (360%) and 21 times (2000%) over that of the spontaneous adhesion of Ti/ABS (no glue) (4.0) and Ti/CF/PC (no glue) (1.0 MPa) [10,11,46].

Therefore, by setting the UTS of Ti/CF/epoxy equal to 1.00, the UTS (*σ*_b_) of Ti/**EB**CF/ABS was increased from a 0.33 to a 0.73 fraction of Ti/CF/epoxy, while the UTS of Ti/**EB**CF/PC was increased from a 0.27 to a 0.82 fraction of Ti/CF/epoxy.

The increase in UTS of the TP joints is attributed to action of HLEBI taking advantage of the high contact surface area of the CFs intricately connected to polymer matrix enhancing adhesion over that of an untreated CF plug to make a stronger joint.

### 3.3. Results for Normalized (Corrected) ^c^*σ*_b_ (^c^UTS) for CFRP Cross-Sectional Area Fraction by Rule of Mixtures

Aircraft fan blades joining Ti with CFRP probably have an entirely CFRP cross-section. Therefore, corrected tensile stress according to the CFRP cross-section (^c^*σ*_b_) was calculated for the samples in our studies by the rule of mixtures [10,11,46]:

^c^*σ*_b,JOINT_ = Σ*n*_i_*σ*_b,i_ = *n*_CFRP_^c^*σ*_b_ + *n*_P_*σ*_b,P_ (3)

where *n*_i_ are fractional cross-sectional surface areas perpendicular to the tensile testing direction for components *i* (in this case, CFRP) and polymer/Ti P, respectively. Rearranging gives [10,11,46]:

^c^*σ*_b_ = [*σ*_b,JOINT_ − *n*_P_*σ*_b,P_]/*n*_CFRP_ (4)

Here, *n*_CFRP_ and *n*_P_ were approximated as 1/13 and 12/13, respectively, according to the specimen geometry in Figure 1.

**Figure 5:**
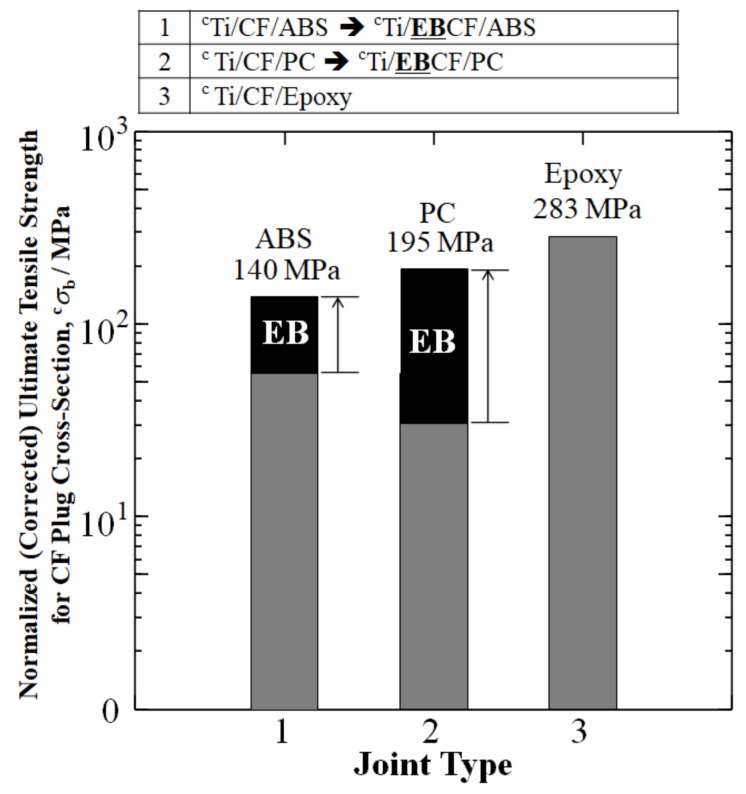
Improvements in corrected (normalized) UTS ^c^*σ*_b_ for CF plug cross-sections of Ti/TP joints by HLEBI for ABS and PC compared with that of untreated Ti/TS epoxy joint. Data is from: Hasegawa, Faudree, Matsumuara, Jimbo, and Nishi (2016) [10]; Hasegawa, Faudree, Enomoto, Takase, Kimura, Tonegawa, Jimbo, Salvia, and Nishi (2017) [11]; and Nishi, Uchida, Faudree, Kaneko, and Kimura (2019) [46], for titanium joints with ABS, PC, and epoxy, respectively. As mentioned earlier, superscript ‘c’ designates normalized (corrected) UTS according to CF portion of cross-section by rule of mixture calculation.

Figure 5 depicts a significant increase in the ^c^*σ*_b_ of TP joints by a 0.30 MGy dose of HLEBI over that of the untreated sample when the cross-sections of the joints were evaluated for the CFRP portion. For untreated ^c^Ti/CF/ABS and ^c^Ti/CF/PC, the ^c^*σ*_b_ were 55 and 30 MPa, respectively. However, applying 0.30 MGy HLEBI to the thermoplastic joints resulted in an increased ^c^*σ*_b_ of 154% (from 55 → 140 MPa) for ^c^Ti/**EB**CF/ABS and 829% (from 30 → 195 MPa) for ^c^Ti/**EB**CF/PC. This was closer to the goal of ^c^*σ*_b_ for thermoset epoxy ^c^Ti/CF/epoxy of 283 MPa.

Therefore, for the CFRP cross-section, by setting the ^c^*σ*_b_ (^c^UTS) of ^c^Ti/CF/epoxy equal to 1.00, the ^c^*σ*_b_ of ^c^Ti/**EB**CF/ABS was increased from a 0.15 to a 0.49 fraction of ^c^Ti/CF/epoxy, while the ^c^*σ*_b_ of ^c^Ti/**EB**CF/PC was increased from a 0.11 to a 0.69 fraction of ^c^Ti/CF/epoxy.

### 3.4. Activation by HLEBI Increasing Adhesion of Carbon Fibers with Thermoplastic

**Figure 6:**
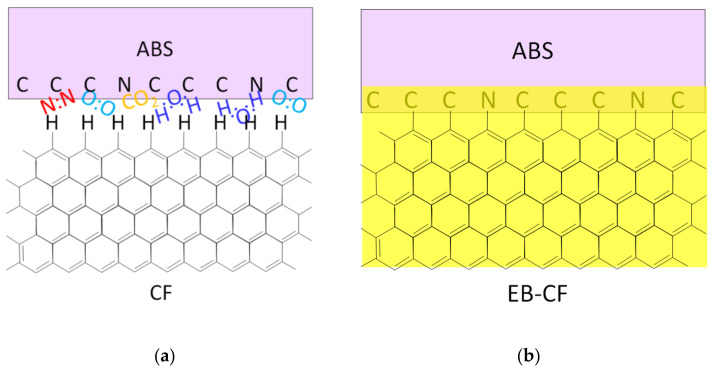
Schematics of (**a**) untreated CF and (**b**) HLEBI-treated CF with ABS thermoplastic. HLEBI activation area is in yellow. Not drawn to scale.

Figure 6a shows that untreated CF exhibits a weak Van der Waals force with thermoplastic ABS with trace N_2_, O_2_, CO_2_, and H_2_O gases in the chamber. On the other hand, as shown in Figure 6b, activating the CF plug with 0.30 MGy HLEBI prior to dipping in the TP resin increased covalent bonding with ABS, raising the UTS of Ti/**EB**CF/TP joints over untreated Ti/CF/TP [10,11]. The HLEBI acted to form active terminated carbon atoms on the surface and activated vacant sites of dangling bonds. ESR studies have shown HLEBI reduces dangling bond density on the CF surface; therefore, the excess charge probably transferred through the highly conductive CFs to the thermoplastic, creating covalent bonds, as depicted in Figure 6b. This reduces fiber pull-out.

Based on the mean density *ρ* (kg/m^3^) of carbon fibers (1760 kg m^−3^) and the irradiation potential at the specimen surface (*V*: keV), the penetration depth *D*th (m) of HLEBI was calculated by the following equation [11,69,70,71]:

*D*th = 66.7*V*^5/3^/*ρ* (5)

to be 123 μm. Since HLEBI was applied to both sides of a CF plug with a thickness 0.23 mm (230 μm), the CF plug was activated throughout its thickness. With a diameter of 6 μm, an extremely high surface area was activated for increased adhesion with the thermoplastic.

## 4. Ti/CF Half-Length: Metallographic Changes Due to Spot Welding

As for the Ti/CF half-length, CF pull-out was not found due to strong adhesion at the Ti–CF interface by the rapid spot welding; hence, fracture occurred in the Ti/Polymer half-length mostly in the form of CF breakage and pull-out [10,11,46].

The rapid spot welding appeared to prevent the excessive formation of embrittling TiC in the Ti/CF half-length with no or minimal damage to the CFs. The XRD results detected trace amounts of crystalline TiC, evidenced by slight peaks at the 2θ of 36, 40, 62, and 76 deg, although sharp TiC peaks were not detected [10,11,45].

Also, TiO_2_ was detected (in pure form, anatase, rutile, and brookite) and reported as small peaks at the 2θ of 30, 35, 41, 57, 60, 69, 75, and 76 deg [11]. TiO_2_ was reported to enhance the interfacial adhesion of Ti/PC and Ti/CF [11].

WDS mapping showed CFs retained their sizes and shapes [11], indicating the rapid spot-welding method acted to minimize high-temperature contact time, preserving the CFs. Furthermore, the Ti was observed to solidify intricately between individual CFs [10,11,45].

### 4.1. Ti/CF Half-Length: Metallographic Process

To describe the metallographic process during rapid heating above the Ti M.P. of 1943 K (1670 °C), a phase of C- and O-rich Ti molten liquid with Ti crystallites was formed from the CF and ~300 ppm trace O_2_ in the EB chamber. Subsequently, during rapid solidification below 1943 K, Ti crystallites containing C and O in Ti molten liquid were formed, although most solids were amorphous or composed of very small crystal grains due to the supercooling. The amorphous structure had advantages over crystal because stress-concentrating grain boundaries were avoided, while reduced grain size increased strength over the larger size. The C- and O-rich Ti molten liquid was in equilibrium with the TiC and TiO_2_ solids formation until total solidification.

**Figure 7:**
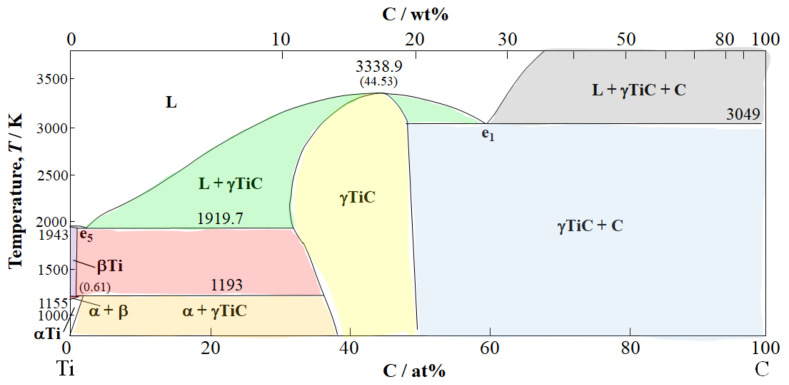
Ti-C phase diagram adapted from Bandyopadhyay, Sharma, and Chakraborti (2000) [72].

Figure 7 illustrates the Ti-C phase diagram [72], which represents the metallographic process during spot welding. A three-phase Ti-C-O would be beneficial; however, as far as the authors know, it was not found in the literature. From XRD analysis, TiC and TiO_2_, along with Ti and C, were detected at the CF–Ti interface layer for the Ti half-length cross-section [10,11,45,46].

The process starts during rapid heating when C contamination up to 1.2 wt.% in the Ti liquid alloy decreases the melting point from 1943 to 1919 K (1670 to 1646 °C) at the eutectic point. In the thin layer around the carbon fibers, higher C contamination from 1.2 to 43 wt.% tremendously enhances the liquidus from 1721 K (1448 °C) to a maximum of 3338.85 K (3065.7 °C) [72]. Hence, Ti solid solutions with possible trace crystalline TiC are formed at the CF–molten Ti interface [45,67].

Moreover, oxygen addition, as evidenced by TiO_2_ peaks by XRD, occurring probably from trace O_2_ in the vacuum chamber elevates the liquidus line to the maximum in the Ti-O phase diagram (not shown) of 2158 K (1885 °C) [73]. The specimens are then put through rapid quench by cold water and solidify in supercooled amorphous form or with small grains. Caution is advised however because, while the joint as a whole is strengthened, excessive contact with the hot molten Ti lowers the strength of CFs [11].

### 4.2. Ti/CF Half-Length: Diffusion

WDS mapping observation of the Ti/CF half-length showed that C atoms were found to diffuse into the Ti matrix, but Ti diffusing into the closely packed hexagonal graphite C structure was not observed [10,11,46]. Additional observation showed C atom diffusion into Ti was particularly around the carbon fiber circumferences in a thin film about ~1 mm thick [4], which probably included the trace TiC detected by XRD.

To discuss the diffusion rates of C, O, and Ti in Ti, the reported diffusion coefficients, *D* (cm^2^/s) are listed in Table 1. It shows that, for α-Ti from 1113 to 873 K (840 to 600 °C), the *D* of C and O cover the same range: for C, 9 × 10^−11^ to 2 × 10^−12^ cm^2^/s; and for O, 0.2 to 2 × 10^−9^ to 6 × 10^−13^ cm^2^/s (although the range for O was wider) [74].

The left column of Table 1 shows that, at higher temperatures above the phase transition of *α* to *β*-Ti, there appears to be a hierarchy of increasing diffusion rate in *β*-Ti: Ti → O → C, with the C atoms having the highest value [74].

If a thin TiC layer was generated at the Ti–CF interface, the diffusion coefficient *D* of the C in TiC was ~5 × 10^−12^ cm^2^/s at ~1723 K (~1450 °C) [75,76]. This was seven orders of magnitude lower than the *D* of C in *β*-Ti of 8 × 10^−5^ cm^2^/s at a similar temperature of 1693 K (1420 °C) (Table 1), indicating that, despite embrittling, the TiC layer can be advantageous for preventing C diffusion into Ti.

Note that the wt.% of O atoms in the Ti crystal structure was reported to slow the diffusion of C in TiC [76].

## 5. Developments in Thermoset Ti/CF/Epoxy Joints

For comparison to Ti/TP joints, the background of developing thermoset Ti/TS/epoxy joints by novel CF plug insert is given here [45,46]. The successful innovation of Ti/CF/epoxy joints could be achieved, but with an epoxy matrix [46]. The spot welding of the Ti/CF half-length and the joint dimensions were identical to those of TPs mentioned above [10,11].

**Figure 8:**
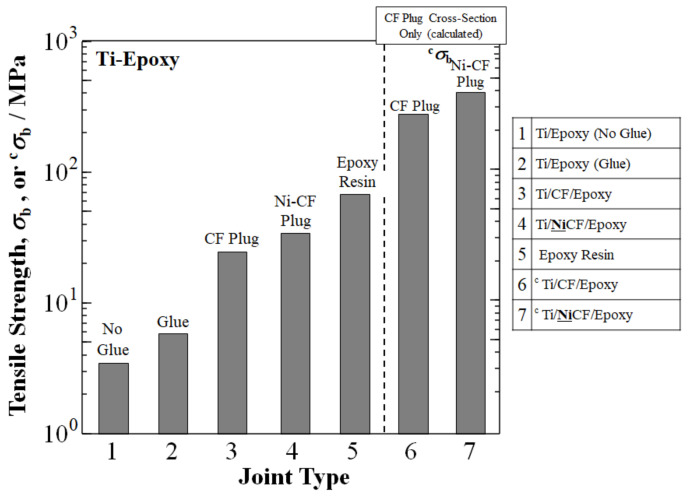
Advances in UTS of Ti/epoxy joints with CF plug. Data is from Hasegawa, Inui, Shiraishi, Ishii, Kasai, Matsumura, and Nishi (2016) [45]; and Nishi, Uchida, Faudree, Kaneko, Kimura (2019) [46]. Reported data of UTS of epoxy resin from Shackelford (2000) is also shown [77].

Figure 8 shows a summary of our research up to now on increasing the UTS of Ti/epoxy joints [45,46]. The UTS of a TS Ti/epoxy spontaneous joint with no glue was shown at *σ*_b_ = 3.5 MPa, while that of Ti/glue/epoxy joint was 5.9 MPa [13]. As mentioned earlier, the CF plug addition boosted the UTS to 25.5 MPa [46]. The Ti/CF/epoxy joint was further strengthened to 35 MPa by a novel process of Ni-coating of the CFs of Ti/**Ni**CF/epoxy joints prior to welding with Ti [46]. This was compared to the UTS of epoxy resin of 69 MPa [77]. However, the CFRP portion of the cross-section for the ^c^Ti/CF/epoxy joint was calculated by the rule of mixtures to be above that of epoxy resin at ^c^*σ*_b_ = 283 MPa. Ni-plating of the CFs prior to welding with Ti for the ^c^Ti/**Ni**CF/epoxy joint increased the ^c^*σ*_b_ further to 45% over the ^c^Ti/CF/epoxy joint with 413 MPa [46].

The rule of mixtures calculation for the CFRP portion of the cross-section indicated that a CF plug can make it possible to increase UTS orders of magnitudes higher than that of Ti/epoxy (no glue) with spontaneous adhesion. The ^c^*σ*_b_ of ^c^Ti/CF/epoxy and ^c^Ti/**Ni**CF/epoxy joints were 80 times (~8000%) and 113 times (~11,700%) larger, respectively, than the *σ*_b_ of Ti/epoxy at 3.5 MPa [46].

### Metallographic Process of Increasing Adhesion by Ni Coating on Ti/CF Half-Length for Ti/**Ni**CF/Epoxy Joints

This paper focuses on increasing the UTS of Ti/TP and Ti/CF/TP joints by HLEBI. However, the metallographic mechanisms for Ti/TS and Ti/**Ni**CF/epoxy joints are briefly covered here.

Although the CF plug greatly enhances UTS over spontaneous adhesion, untreated CF does not bond well due to its inert surface and low wettability, as well as having chemical instability with metals including iron, all of which limit its mechanical properties [78]. Therefore, to raise the UTS of Ti/CF/epoxy joints further, Ni has been used to coat CFs. A Ni plating prevents the encroachment of molten metal at high welding temperatures and excess brittle carbide formation at the CF–metal interface from reactions between carbon and the metal [44]. A Ni coating acts as a buffer with mutual diffusion between Ni and Ti as a gradient absorbing energy during tensile testing to increase strength. As shown in Figure 8, Ni-plated CFs increased the UTS of a Ti/**Ni**CF/epoxy joint to 413 MPa over that of an uncoated Ti/CF/epoxy joint at 283 MPa.

An observation of the Ni-coated area by XRD indicated that trace NiTi and Ni_3_Ti crystallites were formed at the Ni–Ti zone [45] as a diffusion layer in the Ti/**Ni**CF/CFRP joint. The diffusion coefficients for Ti-Ni_3_Ti were from 7.5 × 10^−10^ to 1.8 × 10^−9^ cm^2^/s at 1173 K (900 °C) [79], which were higher than those of the O in *β*-Ti at 0.6 to 1 × 10^−7^ cm^2^/s and the Ti (self-diffusion) in Ti at 6 × 10^−10^ cm^2^/s, both at the same temperature of 1173 K [74]. As mentioned earlier, metallic elements were not detected in the carbon fibers.

## 6. Summary of Our Research Increasing UTS of Ti/TP and Ti/Epoxy Joints

For easy reference, Table 2 and Table 3 give a summary of our research up to now advancing the UTS of hybrid Ti/polymer joints showing: an increase in the UTS (*σ*_b_) (MPa) of treated (HLEBI or Ni) CF plugs over an untreated CF plug condition, as well as UTS improvement over a Ti/polymer (no glue) condition.

Table 2 shows that, for the TPs, Ti/**EB**CF/ABS and Ti/**EB**CF/PC were increased by 114% and 200% over untreated Ti/CF/ABS and Ti/CF/PC and by 355% and 2000% over Ti/ABS and Ti/PC, respectively. For the TSs, Ti/**Ni**CF/epoxy was increased 37% over untreated Ti/CF/epoxy and 900% over Ti/epoxy.

Likewise, Table 3 shows an increase in the normalized (corrected) UTS (^c^*σ*_b_) (MPa) according to the CF portions of the cross-sections. For the TPs, ^c^Ti/**EB**CF/ABS and ^c^Ti/**EB**CF/PC were increased 154% and 829% over untreated ^c^Ti/CF/ABS and ^c^Ti/CF/PC and 3400% and 19,400% over Ti/ABS and Ti/PC, respectively. For the TSs, ^c^Ti/**Ni**CF/epoxy was increased 45% over untreated ^c^Ti/CF/E=epoxy and 11,700% over Ti/epoxy.

**Figure 9:**
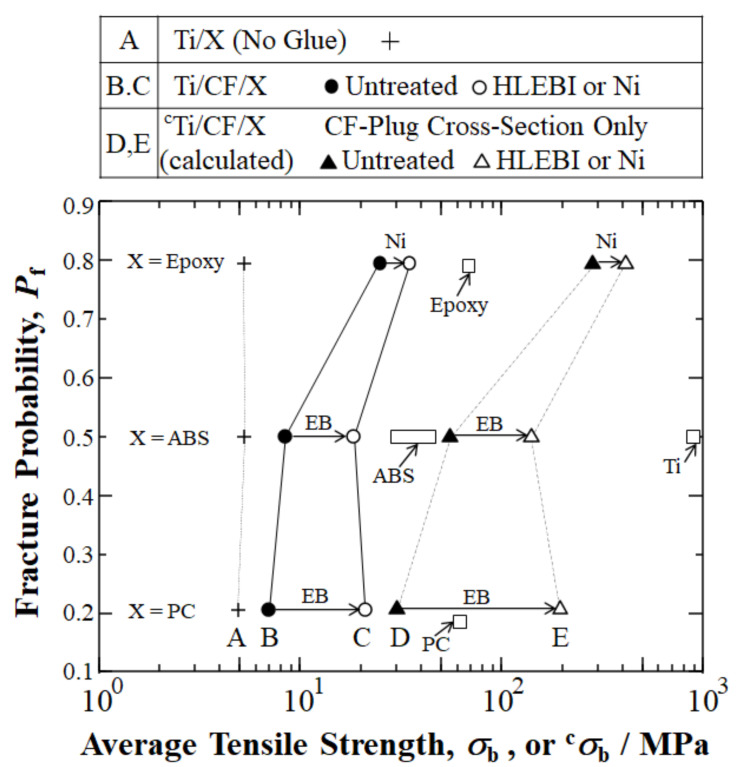
Graphical summary of current status and review showing our previously published results developing Ti/polymer hybrid joints by CF plug insert in terms of fracture probability *P*_f_ vs. reported average UTS *σ*_b_ (MPa). Normalized (corrected) ^c^*σ*_b_ for CF plug cross-sections are shown with data from: Hasegawa, Faudree, Matsumuara, Jimbo, and Nishi (2016) [10]; Hasegawa, Faudree, Enomoto, Takase, Kimura, Tonegawa, Jimbo, Salvia, and Nishi (2017) [11]; and Nishi, Uchida, Faudree, Kaneko, and Kimura (2019) [46], for titanium joints with ABS, PC, and epoxy, respectively, along with *σ*_b_ of bulk Ti from Barksdale (1968) [80] and data of ABS, PC and epoxy from Shackelford (2000) [77].

Figure 9 graphically shows the advances in UTS with reported values for Ti, ABS, PC, and epoxy. In summary, with the experimental data reported, the CF plug appears to tremendously increase the UTS of Ti/polymer joints.

## 7. Conclusions

A review was conducted of the latest studies found in the literature to create strong Ti/CFRP joints. Up to now, methods have included laser treatment, improving bolted joints, carbon fiber nanotubes (CNTs), anodizing, brazing, friction riveting, inductive heating, ultrasonic additive manufacturing, and novel bio-inspired adhesives. However, none of these technologies has applied a carbon fiber (CF) insert intricately embedded into both joint half-lengths for strong adhesive force, taking advantage of the broad interfacial surface area of *d* = 6 μm CF cross-weave cloth. Therefore, we then reviewed our literature on the strengthening Ti/polymer joints by CF plugs by first spot-welding the Ti to CF, followed by dipping the remaining half-length in polymer resin.

Employing a CF plug for Ti/polymer joints resulted in a substantial increase in the ultimate tensile strength (UTS) over spontaneous adhesion without glue: 2.1 times (+113%), 7 times (+600%), and 7 times (+630%) for Ti/CF/ABS, Ti/CF/PC, and Ti/CF/epoxy joints to 8.5, 7.0, and 25.5 MPa, respectively.

However, since thermoplastic polymers (TPs) have poorer adhesion to CF than thermoset (TS) epoxies, CFs were treated with homogeneous electron beam irradiation (HLEBI) prior to dipping in the TP resin. The resulting UTS amounts for acrylonitrile butadiene styrene (ABS) Ti/**EB**CF/ABS and polycarbonate (PC) Ti/**EB**CF/PC joints were increased 114% and 200% to 18.2 and 21.0 MPa, respectively, over those of Ti/CF/ABS and Ti/CF/PC. This was closer to that of the epoxy joint Ti/CF/epoxy at 25.5 MPa. When calculated according to the rule of mixtures (RM) for CF cross-section portions, the UTS of Ti/**EB**CF/ABS and Ti/**EB**CF/PC were increased 154% (from 55 to 140 MPa) and 829% (from 30 to 195 MPa), respectively, over untreated samples, closer to that of Ti/CF/epoxy at 283 MPa.

The strengthening mechanism of the action of HLEBI prevented CF pull-out by apparently creating covalent bonding at the CF–TP interface, as well as growing crystallites into the TP around CF circumference.

Our research employing CF plugs to join Ti and TPs is progressing to reach the ultimate goal of raising the UTS of thermoplastic Ti/CF/TP joints to that of thermoset Ti/CF/epoxy for safety and environmental sustainability.

## Figures and Tables

**Table 1:** Diffusion coefficients for carbon, oxygen, and titanium atoms in titanium. Data is from Nakajima and Koiwa (1991) [74].

Diffusion Coefficients, *D* (cm^2^/s) in Ti			
***D*** **of C in *a*-Ti**		***D*** **of C in *b*-Ti**	
*T* (K)	*D* (cm^2^/s)	*T* (K)	*D* (cm^2^/s)
1113	9 × 10^−11^	1693	8 × 10^−5^
873	2 × 10^−12^	1353	2 × 10^−6^
**D of O in *a*-Ti**		***D*** **of O in *b* -Ti**	
1113	0.2 to 2 × 10^−9^	1693	1 × 10^−6^
873	6 × 10^−13^	1173	0.6 to 1 × 10^−7^
		***D*** **of Ti in Ti (self diffusion)**	
		1693	6 × 10^−8^
		1173	6 × 10^−10^

**Table 2:** Summary of improvements in UTS (*σ*_b_) (MPa) of: treated (HLEBI, or Ni plating) over untreated CF plug joints (left two columns); and total improvement of treated CF plug over Ti/polymer (no plug, no glue) joints (right two columns). Data is from: Hasegawa, Faudree, Matsumuara, Jimbo, and Nishi (2016) [10]; Hasegawa, Faudree, Enomoto, Takase, Kimura, Tonegawa, Jimbo, Salvia, and Nishi (2017) [11]; and Nishi, Uchida, Faudree, Kaneko, and Kimura (2019) [46], for titanium joints with ABS, PC, and epoxy, respectively.

Treated CF-Plug over		Treated CF-Plug over Ti/Polymer (No Glue) Joints	
Untreated CF-Plug Joints	*s*_b_ (MPa)	Joint	*s*_b_ (MPa)
Ti/CF/ABS	8.5	Ti/ABS (No Glue)	4
Ti/**EB**CF/ABS	18.2	Ti/**EB**CF/ABS	18.2
imp.	114%	imp.	355%
Ti/CF/PC	7	Ti/PC (No Glue)	1
Ti/**EB**CF/PC	21	Ti/**EB**CF/PC	21
imp.	200%	imp.	2000%
Ti/CF/Epoxy	25.5	Ti/Epoxy (No Glue)	3.5
Ti/**Ni**CF/Epoxy	35	Ti/**Ni**CF/Epoxy	35
imp.	37%	imp.	900%

**Table 3:** Summary of improvements in normalized (corrected) values of UTS (^c^*σ*_b_) (MPa) for CF cross-section portions of joints calculated according to rule of mixtures in Table 2 for: treated (HLEBI or Ni plating) joints over untreated CF plug joints (left two columns); and total improvement of treated CF plug joints over Ti/polymer (no plug, no glue) joints (right two columns). Data is from: Hasegawa, Faudree, Matsumuara, Jimbo, and Nishi (2016) [10]; Hasegawa, Faudree, Enomoto, Takase, Kimura, Tonegawa, Jimbo, Salvia, and Nishi (2017) [11]; and Nishi, Uchida, Faudree, Kaneko, and Kimura (2019) [46], for titanium joints with ABS, PC, and epoxy, respectively.

Treated CF-Plug over		Treated CF-Plug over [Ti/Polymer] (No Glue) Joints	
Untreated CF-Plug Joints	^c^*s*_b_ (MPa)	Joint	^c^*s*_b_ or *s*_b_ (MPa)
^c^Ti/CF/ABS	55	Ti/ABS (No Glue)	4
^c^Ti/**EB**CF/ABS	140	^c^Ti/**EB**CF/ABS	140
imp.	154%	imp.	3400%
^c^Ti/CF/PC	21	Ti/PC (No Glue)	1
^c^Ti/**EB**CF/PC	195	^c^Ti/**EB**CF/PC	195
imp.	829%	imp.	19,400%
^c^Ti/CF/Epoxy	283	Ti/Epoxy (No Glue)	3.5
^c^Ti/**Ni**CF/Epoxy	413	^c^Ti/**Ni**CF/Epoxy	413
imp.	45%	imp.	11,700%

## Data Availability

The data presented in this study are available on request from the corresponding author. At the time the project was carried out, there was no obligation to make the data publicly available.
